# The integration of signaling and the spatial organization of the T cell synapse

**DOI:** 10.3389/fimmu.2012.00352

**Published:** 2012-11-23

**Authors:** Jérémie Rossy, David J. Williamson, Carola Benzing, Katharina Gaus

**Affiliations:** Centre for Vascular Research and Australian Centre for Nanomedicine, University of New South WalesSydney, NSW, Australia

**Keywords:** T cell receptor, membrane organization, receptor oligomerization, signaling assembly, T cell activation

## Abstract

Engagement of the T cell antigen receptor (TCR) triggers signaling pathways that lead to T cell selection, differentiation and clonal expansion. Superimposed onto the biochemical network is a spatial organization that describes individual receptor molecules, dimers, oligomers and higher order structures. Here we discuss recent findings and new concepts that may regulate TCR organization in naïve and memory T cells. A key question that has emerged is how antigen-TCR interactions encode spatial information to direct T cell activation and differentiation. Single molecule super-resolution microscopy may become an important tool in decoding receptor organization at the molecular level.

## TCR signaling

**Figure 1:**
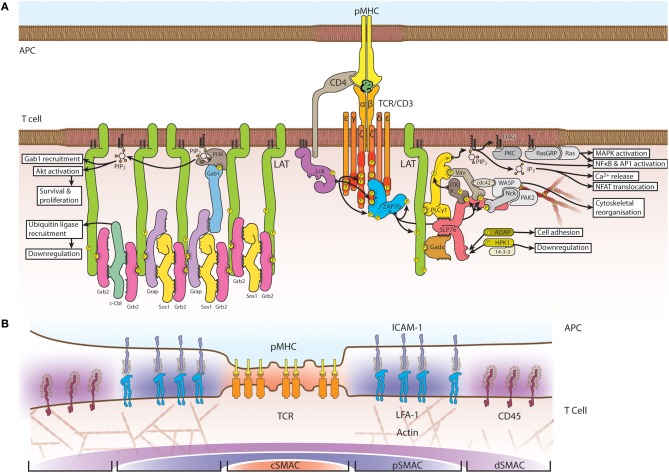
**Schematic structure of the TCR signaling assembly and immunological synapse. (A)** Engagement of TCR ligation and co-receptors, such as CD4 and CD28, recruits Lck, which phosphorylates CD3 at immunoreceptor tyrosine-based activation motifs (ITAMs). ZAP70 binds double-phosphorylated ITAM residues whereupon it is phosphorylated and activated by Lck. LAT is recruited to the activated TCR complex and is phosphorylated by ZAP70 at several tyrosine residues on its C-terminal tail. LAT phosphorylation induces the assembly of signaling adapters and effectors; multi-point binding options between LAT, Grb2, Sos1 and Grap facilitate the formation of an extended protein network. LAT-nucleated signaling also occurs through Gads and SLP76. **(B)** At larger scales, the immunological synapse forms into distinct supramolecular activation clusters (SMACs), with the central SMAC (cSMAC, orange) rich in TCRs. Surrounding this layer is the peripheral SMAC (pSMAC, blue) enriched in signaling cofactors and adhesion complexes. A further region, the distal SMAC (dSMAC, purple) comprises glycoproteins such as CD45 and CD44.

Activation of T cells is a key element in adaptive immunity and requires the coordination of highly complex signal transduction networks (Figure 1A). The process begins when the T cell receptor (TCR) binds to peptide-loaded major histocompatibility complexes (pMHC) (Huppa and Davis, 2003; van der Merwe and Dushek, 2011). While the TCR's peptide-recognizing αβ heterodimer has no intrinsic catalytic activity, it forms a multi-molecular complex with the dimers CD3εγ, CD3εδ, and CD3ζζ, which have long cytoplasmic domains containing immunoreceptor tyrosine-based activation motifs (ITAMs) (Call et al., 2004). For signaling to proceed, it is necessary that at least two ITAMs are phosphorylated by the Src family kinase lymphocyte-specific protein tyrosine kinase (Lck) that is anchored to the inner leaflet of the plasma membrane (Palacios and Weiss, 2004). Curiously, a proportion of Lck is already activated in resting cells and there is no evidence for TCR or co-receptor induction of Lck activity (Paster et al., 2009; Nika et al., 2010) so it is currently not clear how Lck distinguishes between non-engaged and engaged TCR (Zhang et al., 2011). One possibility is the spatial segregation of TCR and Lck from phosphatases such as CD45 (Davis and Van Der Merwe, 2006; Rossy et al., 2012). Phosphorylated ITAMs serve as recruitment and activation sites for zeta chain-associated protein kinase of 70 kDa (ZAP-70), whose activity is essential in conventional T cells but not in regulatory T cells (Au-Yeung et al., 2010).

The next step in the signaling cascade is the recruitment and phosphorylation of linker for activation of T cells (LAT) that is essential for TCR signaling (Finco et al., 1998), T cell activation (Zhang et al., 1999a) and development (Zhang et al., 1999b). LAT serves as a platform for several adapter and effector molecules (Figure 1A) including phospholipase C gamma (PLCγ) (Zhang et al., 2000), phosphatidylinositol 3-kinase (PI3K) (Cruz-Orcutt and Houtman, 2009) Src-homology-2-domain-containing leukocyte protein of 76 kDa (SLP76) (Wu and Koretzky, 2004), growth factor receptor-bound protein 2 (Grb2) (Zhang et al., 2000) and the Grb2-homologous adapter (GADS) (Zhang et al., 2000; Liu et al., 2001). The mechanism of LAT recruitment is controversial, as discussed below, and it has been suggested that there are LAT-dependent and -independent signaling pathways (Malissen and Marguet, 2011).

Ultimately, TCR activation-induced signaling cascades result in actin cytoskeleton restructuring and induction of gene expression and cytokine secretion. Whether the signals from various pathways are integrated downstream so that the different arms of the signaling process function as independent controls or whether hierarchies exist where specific signaling signatures dominate others is currently not known. Here we focus on signaling molecules up to and including LAT, as each of these steps in early TCR signaling are essential.

## TCR microclusters and the immunological synapse

The interface between a T cell and an antigen-presenting cell (APC) is referred to as the immunological synapse (Huppa and Davis, 2003; van der Merwe and Dushek, 2011). In the original model, the synapse is organized into supramolecular activation clusters (SMACs) comprising three distinct concentric regions (Figure 1B): a central region, designated as the cSMAC, contains a high number of TCRs. This layer is surrounded by the peripheral region, the pSMAC, which is enriched in adhesion proteins such as leukocyte function-associated antigen 1 (LFA1) and talin. The third and outermost region is the distal SMAC (dSMAC), which contains actin and CD45 (Monks et al., 1998; Grakoui et al., 1999). This classic bull's-eye pattern of the immunological synapse emerges from the dynamic interactions of the TCR with its signaling partners. After initial contact, TCR engagement triggers the formation of TCR microclusters of the proteins Lck, ZAP70, LAT, and SLP76 (Bunnell et al., 2002, 2006). In a mature synapse with a fully formed cSMAC and pSMAC, TCR microclusters continuously form in the pSMAC (Campi et al., 2005; Varma et al., 2006) and are transported to the cSMAC in an actin- and myosin-dependent manner (Kaizuka et al., 2007; Ilani et al., 2009); the cSMAC is also where signaling is thought to be terminated and the receptor internalized (Liu et al., 2000; Coombs et al., 2002). The dynamic spatial organization of the synapse, as observed mainly on supported lipid bilayers, influences signaling activity and *vice versa*. The formation of TCR microclusters and initiation of signaling precede the formation of the cSMAC and initially occur throughout the entire T cell-APC contact area (Lee et al., 2002; Yokosuka et al., 2005). Additionally, the phosphatase CD45 is excluded from TCR microclusters in immature synapses (Varma et al., 2006) and from the cSMAC where phosphorylated TCRs accumulates in fully established synapses (Leupin et al., 2000), suggesting that segregation of the receptor from CD45 is mechanistically linked to receptor phosphorylation.

The bull's eye pattern of mature synapses was originally observed in helper T cells but has since been described for cytotoxic T cells (Anikeeva et al., 2005), regulatory T cells (Zanin-Zhorov et al., 2010), B cells (Depoil et al., 2008; Randall et al., 2009) and natural killer (NK) cells (McCarthy et al., 2007). Surprisingly, a SMAC organization is not required for T cell signaling. T cell interfaces with dendritic cells (Brossard et al., 2005) and Th2 cells (Thauland et al., 2008) result in multiple focal structures lacking the SMAC architecture. Naïve T cells in the lymph node form TCR clusters independent of antigen presentation while the dominant feature observed in the presence of antigen was TCR internalization, which was also not contingent on cSMAC formation (Friedman et al., 2010). In a detailed study, Schubert et al. recently examined the patterns of immunological synapses in self-reactive T cells clonally derived from patients with multiple sclerosis and type 1 diabetes (Schubert et al., 2012) and found that despite strong TCR phosphorylation and signaling activity, essentially no cSMAC was formed in self-reactive T cells.

While cSMACs may not be required for signal initiation (Lee et al., 2002), they appear to function in signal modulation and termination. TCR microclusters are actively transported toward the cSMAC where they co-localize with markers of protein degradation such as LBPA (Varma et al., 2006) and components of the ubiquitin pathway (Vardhana et al., 2010). Internalization of TCR bound to pMHCs at the cSMAC could be mediated by phagocytic processes (Alarcón et al., 2011). Although TCR signaling of strong agonist is terminated at the cSMAC, weak activation results in TCR signaling within the cSMAC (Cemerski et al., 2008), thus acting as a signal modulator. A further role for the SMACs may be in focused secretion of lysosomes from cytotoxic T cells to virally infect and tumor cells or of cytokines to antigen presenting cells (Griffiths et al., 2010).

The underlying mechanisms of synapse patterning and TCR microcluster formation are still not fully understood. Both, inhibition of actin flow and myosin-II activity impair TCR microcluster and synapse formation (Campi et al., 2005; Kaizuka et al., 2007; Ilani et al., 2009). Recently, it has been suggested that under subtle perturbation of actomyosin dynamics (rather than complete inhibition of the network), actin retrograde flow is the main driver for TCR microcluster accumulation in the cSMAC (Babich et al., 2012). Interestingly, an intact actin cytoskeleton is required for initial TCR microclusters formation but, once established, TCR microclusters are sufficiently stable without a functional actin network. Hence actin and acto-myosin contraction are only required at the early stages of synapse formation. *In vivo*, the T cell-APC contact zone is fluid due to the T cells motility (Mempel et al., 2004; Miller et al., 2004) and immunological synapses are not as stable as in cells activated on bilayers. The duration of these transient T cell-APC interactions may determine the signaling switch between tolerance and activation (Katzman et al., 2010). Further, in motile synapses, the movement of TCR microclusters is aligned with the cell migration and not oriented toward the cSMAC. Both TCRs and cSMAC are consistently relocated to actin-poor regions that required local actin depolymerisation (Beemiller et al., 2012). Whether cortical actin is simply a means to compartmentalize the T cell membrane, as proposed in the “picket-fence” membrane model (Kusumi et al., 2011), or plays a more active role in TCR cluster formation remains to be seen.

An association of signaling proteins with protein networks (Douglass and Vale, 2005) and membrane domains (Viola et al., 1999; Janes et al., 2000) has been proposed as an underlying mechanism for the lateral organization of the plasma membrane. Indeed the membrane environment at T cell activation sites is considerably more ordered than in resting cells (Gaus et al., 2005; Owen et al., 2010) and biochemically resembles lipid rafts due to cholesterol and sphingomyelin enrichment (Zech et al., 2009). In addition, preventing membrane condensation resulted in fewer TCR microclusters at the cell surface and impaired signaling and activation responses (Rentero et al., 2008). However, whether the protein affinity for this membrane environment is sufficient to drive protein sorting and clustering is still unknown. The lipid anchor of Lck, for example, does not control Lck distribution and diffusion (Douglass and Vale, 2005), lipid raft reporters are not associated with TCR microclusters (Hashimoto-Tane et al., 2010) and do not cluster upon TCR activation (Glebov and Nichols, 2004). Furthermore, the two palmitoylation groups on LAT are mainly responsible for delivery of the protein to the plasma membrane (Tanimura et al., 2006; Hundt et al., 2009) rather than imposing an association to lipid raft domains (Zhang et al., 1998; Lin et al., 1999). Although the contribution of lipid rafts to TCR signaling remains controversial, lipids clearly play a role in T cell activation (Geyeregger et al., 2005; Galli and Calder, 2009). In addition, a specific membrane environment may stabilize TCR microclusters (Choudhuri and Dustin, 2010) and control the interaction of basic residue-rich stretches in the ITAM domains with the plasma membrane (Zhang et al., 2011).

## New models for LAT signaling

**Figure 2:**
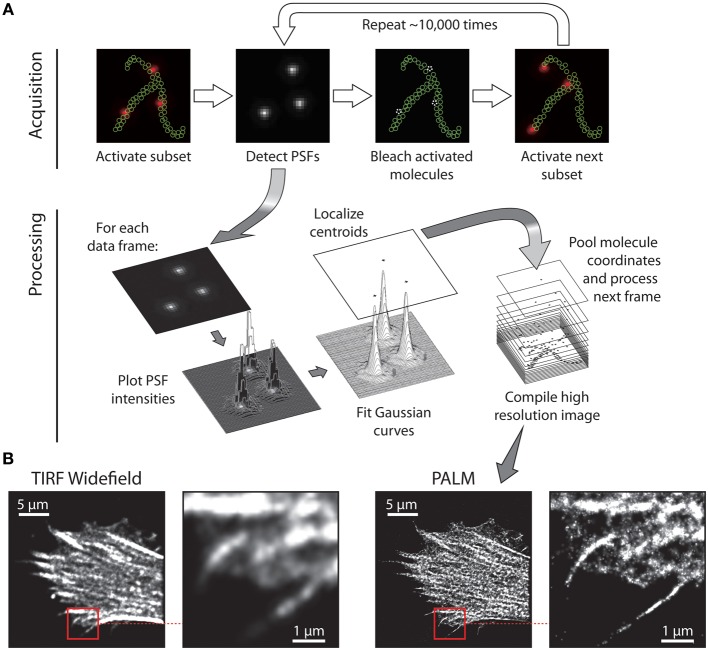
**Principle of single molecule localization microscopy. (A)** Imaging sequence for data acquisition in which a spare subset of molecules is activated, imaged, and bleached. Following acquisition of the raw PSF data, the image sequence is processed to localize each molecule's point-spread function (PSF) with nanometre precision by fitting Gaussian distributions to the intensity profile of each molecule. By repeating photo-activation imaging and fitting for typically 15,000–20,000 frames, a coordinate map of all detected molecules is generated. **(B)** Example data: HeLa cell expressing Lifeact (an F-actin binding protein) fused to the photoswitchable protein tdEos, observed with TIRF, widefield microscopy and then with TIRF PALM. The substantial increase in resolution is evident by comparing the zoomed TIRF and PALM regions, indicated by the red boxes.

Insights into the spatial organization of immunological synapses have been made possible by total internal reflection fluorescence (TIRF) microscopy and the use of supported planar lipid bilayers within which adhesion and MHC molecules are laterally mobile. More recently the exquisite signal-to-noise ratio of TIRF microscopy has been exploited for super-resolution techniques, namely photoactivated localization microscopy (PALM) (Betzig et al., 2006; Hess et al., 2006) and stochastic optical reconstruction microscopy (STORM) (Rust et al., 2006) that can localize individual proteins molecule in intact cells with nanometre precision (Table 1). PALM and STORM [and its derivative direct STORM or dSTORM (Heilemann et al., 2008)] achieve high imaging resolution by employing switchable fluorescent signals (Figure 2). By controlling the fluorescence of labeled molecules from dark to bright states, individual molecules are temporally separated, and thus identified, from their unswitched neighbors within a small, diffraction-limited, spatial area. The practical execution of these techniques requires fluorescent proteins (in the case of PALM) or organic dyes (for STORM and dSTORM) which are able to transition from dark to bright states (photoactivation) or from one emission spectra to another (photoswitching) when irradiated with a specific switching or activation laser, usually operating at a sufficiently low power to ensure only a few molecules in the population are driven into the switched fluorescent state. Once a sparse set of molecules are switched, they can be excited into fluorescence by a much higher power imaging laser. In the case of PALM, the high intensity of the excitation laser is usually sufficient to destroy the protein (or at least its chromophore) through photobleaching, thus removing it from the total pool of labeled molecules. For STORM and dSTORM, the high intensity activation laser drives the dye into a dark state, from which it can be recovered by the activation laser for multiple fluorescence cycles before photobleaching. This cycle of photoactivation, fluorescence emission, and photobleaching is repeated until all the labeled molecules have been registered. The fluorescence intensity profile, known as the point-spread function (PSF) of each individual molecule is analyzed to determine the localization coordinates for each molecule. The fitting process also returns the localization precision and number of photons emitted from each molecule.

**Table 1:** **Advantages and limitations of TIRFM, PALM, and STORM**.

**Acronym**	**Name**	**Description**
TIRFM	Total Internal Reflection Fluorescence Microscopy	Principle of operation: Directing an excitation source at a critical angle to the glass coverslip, such that the beam is totally internally reflected, generates an evanescent wave penetrating approximately 100 nm into the sample. Fluorophores within this range are excited whereas material deeper in the sample will remain dark, effectively eliminating out-of-focus fluorescence, including autofluorescence.
		Advantages Minimal background signal; increased signal-to-noise. Tight field depth, corresponding to the evanescent field penetration.
		Limitations: Subject to the diffraction limit. Only samples which are adjacent to the glass-water interface can be accessed.
PALM	Photo-Activatable Localization Microscopy	Individual photoswitchable or photoactivatable proteins are converted, at very low frequency, into the imaging channel. These sparse, switched molecules are then excited, their spatial positions localized, and bleached. Thousands of successive rounds of switching/activation, excitation, and bleaching are performed to generate a map of all the molecule positions.
		Advantages: High resolution, single molecule localization to 20–50 nm in XY. Excellent labeling specificity conferred by fusion proteins. Compatible with live cell imaging. Easily adapted into 3D with additional optics.
		Limitations: Long acquisition and processing times. Poor photon yield from fluorescent proteins decreases molecule localization precision. Care must be taken to avoid transfection and over-expression artefacts. Endogenous proteins cannot be studied.
STORM	STochastic Optical Reconstruction Microscopy	The same principle as for PALM with conventional dyes conjugated to antibodies as fluorophores.
		Advantages: High resolution, single molecule localization to 20–50 nm in XY. Conventional immunofluorescence dyes can be used. Endogenous proteins can be studied, including modified (e.g. phosphorylated) proteins. Easily adapted into 3D with additional optics.
		Limitations: Long acquisition and processing times. Less compatible with live-cell imaging. Care must be taken to avoid fixation and staining artefacts.

**Figure 3:**
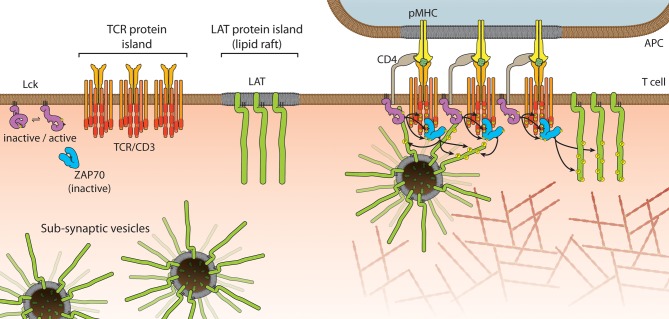
**Models of LAT recruitment.** Protein islands in the T cell membrane can aggregate or coalesce during TCR activation to facilitate phosphorylation and signal transduction. An alternative model invokes sub-synaptic vesicles, which could be signaling endosomes, which translocate to sites of activated TCR during signal transduction. The two models are not mutually exclusive.

Mark Davis and his team used PALM and electron microscopy to put forward the model that the TCR and LAT are segregated in sub-micrometer “protein islands” (Figure 3) that coalesce, but do not mix, upon receptor activation (Lillemeier et al., 2010). The notion of such islands comes from their previous work showing that protein-rich domains are surrounded by a protein-poor “lipid sea” on the plasma membrane (Lillemeier et al., 2006). The implication of this model is that an insulating layer exists around the receptor and LAT islands, which needs to be overcome in order for signaling to be initiated (Dustin and Depoil, 2011). We also used PALM to quantify LAT clustering but came to a very different conclusion (Williamson et al., 2011). Unexpectedly, we found a 2.7-fold increase in the number of LAT molecules at the TCR activation site, which were not laterally recruited from non-activated areas of the plasma membrane. When surface-expressed LAT was bound to streptavidin-coated beads outside the activation zone, LAT recruitment and phosphorylation was normal, clearly indicating that an intracellular pool of LAT is sufficient to drive signaling under these conditions. The existence of LAT sub-synaptic vesicles (Figure 3) was previously demonstrated but whether LAT vesicles are phosphorylated in *trans* and act as signaling endosomes or whether LAT vesicles stay tethered to, or even fuse with, the plasma membrane is currently not known. The two models of pre-existing clusters or islands of LAT and LAT vesicles are not mutually exclusive (Figure 3). In addition to the linear signaling pathway of TCR→Lck→Zap70→LAT→SLP76 that is viewed to take place at the plasma membrane, an alternative pathway may exist in which LAT vesicles dock to the plasma membrane at sites of SLP76-GADS complexes (Purbhoo et al., 2010). Support for this second pathway comes from genetic studies in which LAT was deleted in CD4+ T cells after thymic selection (Mingueneau et al., 2009). Not only did these LAT-deficient CD4+ T cells respond to TCR engagement with Lck and ZAP70 phosphorylation of their targets including SLP76, they fully recapitulated the lymphoproliferative disorders associated with constitutive LAT mutation. Hence SLP76 can participate in T cell signaling independently from LAT (Malissen and Marguet, 2011). If in wild-type T cells the two pathways coexist, it will be interesting to see whether the manner of TCR activation (number of engaged TCR, peptide affinity, off/on rates etc.) selects one pathway over the other; if there is synergy or redundancy between the two pathways, and whether this leads to differential signaling outcomes. In this context it is interesting to note that phosphorylation of LAT occurs within 4 s of TCR-pMHC engagement and calcium fluxes after 6–7 s but while diacylglycerol production is strongly desensitized shortly after TCR activation, LAT phosphorylation is not (Huse et al., 2007). It is also possible that the pre-existing compartmentalization of LAT into membrane domains and vesicles determines TCR signal strength, signal maintenance, and/or contributes to T cell specialization.

## Spatial organization of TCRs

The report of TCR islands (Lillemeier et al., 2010) has been unexpected since previous studies described a different spatial organization of the TCR. Using a fluorescence technique called dynamic single-molecule colocalization (Dunne et al., 2009), a form of single-molecule spectroscopy, David Klenerman, Simon Davis and colleagues showed that the αβ heterodimer of the TCR/CD3 complex is essentially monomeric (James et al., 2011). These measurements were taken at the apical surface of T cells that is not in contact with the glass coverslip or supported lipid bilayer, and activation of TCR occurred through soluble agonists. Whether the experimental conditions account for the differences in TCR organization remains to be seen. Alternatively, individual TCRs may diffuse freely within TCR islands that are positionally stable (Lillemeier et al., 2010). Indeed, James et al. commented that single αβ heterodimers exhibit non-random confinement (James et al., 2007). By what mechanism such confinement occurs will be important to know because it not only determines the compartmentalization of the receptor but also which interactions are available following receptor engagements. Given the mobility of the receptor, it is unlikely that high affinity protein-protein interactions are responsible for confinement, as previously proposed of LAT and Lck (Douglass and Vale, 2005). Alternative mechanisms of receptor confinement are similar to those explored in the context of TCR microclusters, i.e., membrane domains and restrictions imposed by the underlying, membrane-proximal cytoskeleton.

To which extent does the spatial organization of the TCR contribute to T cell signaling and function? This intriguing question has been asked by many researchers but is experimentally difficult to examine. In a comprehensive analysis of four different class II- and I-restricted TCR transgenic mouse models, Prutic et al. found receptor clustering was only important in one specific low affinity/avidity T cell system where TCR accumulation at the cSMAC facilitated integration with costimulatory signals (Purtic et al., 2005). When cSMAC/pSMAC patterns are disturbed by placing T cells on lipid bilayers that are themselves compartmentalized by metal lines or grids, T cell signaling can be prolonged because TCRs cannot accumulate in the cSMAC or be down-regulated as co-receptors are segregated from the TCR (Manz and Groves, 2010). An alternative approach is to target the expression of downstream signaling proteins that do not directly impact on early TCR signaling events. The loss of the tyrosine kinase interleukin-2 (IL2)-inducible T cell kinase (Itk) resulted in unusual spatial organization of the immunological synapse with a mislocalization of the Rho GTPase CDC42 and a concomitant loss of actin accumulation at the synapse (Singleton et al., 2011). These findings illustrate that a downstream signaling molecule can influence the synapse organization of upstream signaling proteins, suggesting that the non-linearity of signaling pathways is interlinked with protein trafficking and membrane compartmentalization at multiple stages.

In 2011, two very different papers were published that we believe will become landmark publications. Firstly, Manz et al. used supported lipid bilayers on metal grids to control the number of peptides that can cluster together without altering the total number of peptides engaged by the T cell (Manz et al., 2011). It was therefore possible to determine how TCR clustering supports the astonishing sensitivity of T cells, which can respond to even a few agonists peptide molecules (Sykulev et al., 1996; Irvine et al., 2002). Limiting TCR clustering at a fixed total pMHC density indeed reduced T cell sensitivity and the probability of intracellular calcium fluxes (Manz et al., 2011). Such stochastic analysis revealed an activation threshold for the number of activating ligands per individual TCR cluster and not per cell, with a minimum of four pMHC in a signal cluster required for calcium signaling. Similar to CD4 blocking which increases the number of peptides required to initiate a T cell responds several fold (Irvine et al., 2002; Krogsgaard et al., 2005), this study suggests that costimulation of CD28 could lower the ligands-per-cluster threshold. In the second study that we would like to highlight, Kumar et al. established a link between the ability of TCR to form oligomers (Schamel et al., 2005; Lillemeier et al., 2010) and the T cell response to antigen stimulation (Kumar et al., 2011). Previously stimulated T cells displayed larger TCR oligomers at their surface than naïve cells and the increased sensitivity of experienced and memory T cells correlated with a higher level of TCR oligomerization. Importantly, a point mutation in the transmembrane domain of CD3ζ involved in tetramer formation (Torres et al., 2002) resulted in a diminution in TCR oligomers and a concomitant decrease in the TCR response to stimulation (Kumar et al., 2011). Hence TCR clustering could be responsible for setting the TCR activation threshold and a key discriminating factor between naïve and memory T cells.

## Does TCR affinity and quaternary structure contribute to TCR triggering?

One of the key features of the T cell system is that ligand-receptor interactions occur on the 2-dimensional surfaces of cell membrane. Comparing 2D affinities of TCR-pMHC binding kinetics with 3D affinities in solution revealed unexpected results. By assuming that the TCR and its ligand fully access the whole inter-membrane space, Huppa et al. showed that the association rate measured in 2D was 100 fold faster than the one measured in 3D (Huppa et al., 2010). Using a micropipette and a biomembrane force probe to quantify the deformation of a red blood cell or the thermal fluctuation of a bead that were both functionalized with pMHC, Huang et al. observed that 2D affinities had a broad range over a panel of pMHCs that matched T cell proliferation responses (Huang et al., 2010). Association and disassociation rates were both significantly faster in these 2D assays compared to 3D solution measurements. In solution, dissociation rates were the best predictor for T cell responses suggesting that slow pMHC dissociation induces T cell activation. Conversely, in the 2D scenario, it was the extremely fast association rates that drove TCR-pMHC responses. This opens the possibility that rapid antigen sampling and possibly serial engagement, where a few pMHC are repeatedly engaged by the same TCRs or TCRs within the same cluster (Aleksic et al., 2010) are mechanisms by which the high concentration of self-pMHC background is overcome *in vivo*. The efficiency of serial TCR-pMHC engagements would be enhanced by a non-random distribution of TCRs and the relative immobility to TCR clusters or islands (Lillemeier et al., 2010), simply because dissociated pMHC can be recaptured by neighboring TCRs. This model was supported by a single-molecule fluorescence resonance energy transfer (smFRET) study where the duration of TCR-pMHC interactions was driven by the high on-rate (Huppa et al., 2010). Interestingly, the authors showed in the same study that blocking CD4 engagement with antibodies did not alter TCR-pMHC binding. In self-reactive T cells that failed to form synapses and did not accumulate TCR in the cSMAC, the off-rates of TCR-pMHC binding were normal while the on-rates were significantly slower compared to TCRs binding corresponding peptides of viral-specific T cells (Schubert et al., 2012), further lending weight to the TCR-pMHC serial engagement model. However, recently it was shown that serial engagement of TCRs is not necessary for activation when pMHC monomers are cross-linked to TCRs (Xie et al., 2012).

**Figure 4:**
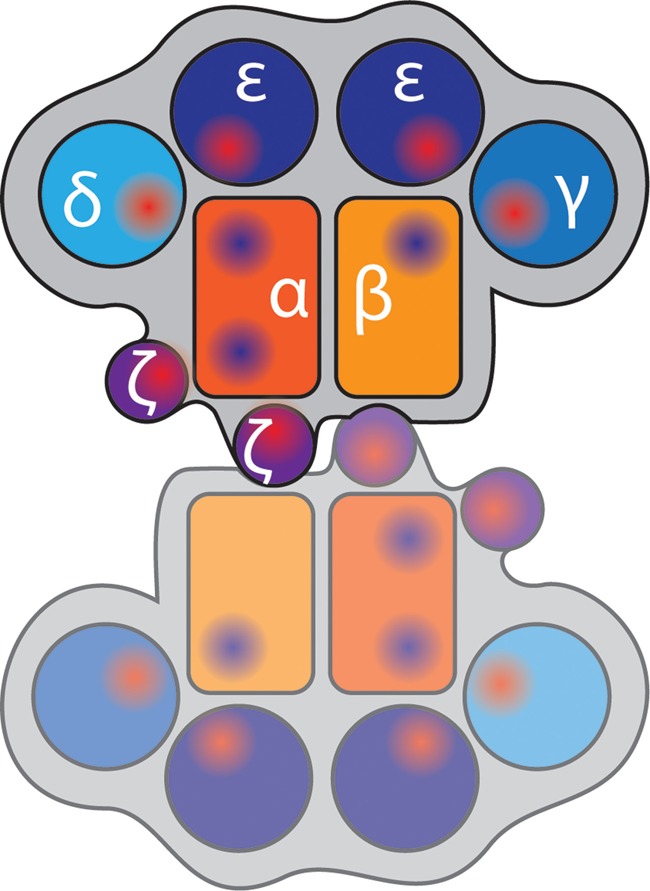
**Model of TCR complex dimerization.** Heterodimers of TCRα and TCRβ (orange and yellow) are opposed due to the short ectodomains of CD3ζ (purple), which places CD3γ, CD3δ, and the two CD3ε chains (shades of blue) around the outside. The shapes represent immunoglobulin domains of the components; and red and blue shaded regions indicate positive and negatively charged regions, respectively.

Are there any clues in the TCR structure and pMHC binding topography that tell us about the arrangements of αβ heterodimer with the CD3 dimers (i.e., quaternary structure) or whether a TCR forms dimers and higher-order oligomers? Like immunoglobulin (Ig) Fab fragments, the αβ TCR heterodimer has subunits consisting of one variable (*V*) and one constant (*C*) Ig domain in the extracellular segment. However, unlike antibodies, there is an elongation connecting the F and G β-strands in the Cβ domain, called the Cβ FG loop (Wang et al., 1998). This structural feature is conserved in all mammalian αβ TCRs studied to date and probably co-evolved with the development of distinct CD3δ and CD3γ genes (Kim et al., 2010). It has been proposed that the Cβ FG loop enforces a high level of rigidity to the αβ heterodimer, and consequently the structure of the TCR complex does not undergo major conformational changes upon pMHC binding (Rudolph et al., 2006). Although the Cβ FG loop is not involved in antigen binding, its deletion impairs cytokine production and T cell proliferation upon receptor stimulation. CD3ε, CD3γ, and CD3δ have each a single extracellular Ig domain while CD3ζ has essentially no extracellular domain. All CD3 units have negatively charged residues in the transmembrane domain that drives paring of the dimers—CD3εδ, CD3εγ, and CD3ζζ—with TCRα and TCRβ because the paired acidic domains can interact with positive charges at the same depth in the transmembrane regions of the TCR (Call et al., 2010). The CD3εγ and CD3εδ dimers have a highly conserved hydrophobic interface and adopt a side-by-side configuration (Sun et al., 2004). A model of the entire TCR complex (Figure 4) has evolved from the topography of the heavily glycosylated ectodomains of αβ, CD3εγ, and CD3εδ and recapitulates the known chain association with CD3ε-CD3γ-TCRα-CD3ζ-CD3ζ as one cluster and CD3ε-CD3γ-TCRβ as a second cluster (Sun et al., 2004). Mark Davis and colleagues engineered a dimerization reporter system—based on the erythropoietin receptor—that only signals and drives cell proliferation when signaling domains are juxtaposed (Kuhns et al., 2010). They showed that the CD3 heterodimers are assembled in tandem on one side of the αβTCR, and leave the other side free to interact with other αβTCR units. Kai Wucherpfenning and his colleagues placed the CD3ζζ on the other side of the αβ TCR (Call et al., 2002, 2006), and as CD3ζ lacks an extracellular domain, this arrangement leaves one side of the αβ TCR open for dimerization (Figure 4). Importantly, the Cβ FG is in close proximity with one of the CD3ε units and therefore does not prevent dimerization. Mutations at the αβ TCR “dimerization interface” in the AB loop, C and F strand in Cα of TCRα-transmembrane domain (TM) and TCRβ-TM chimeras slightly impaired calcium fluxes and severely impacted on TCR accumulation in the cSMAC (Kuhns et al., 2010) indicating that TCR dimerization is important for synapse organization. These findings are interesting because TCR dimerization has been previously proposed as a mechanism to initiate signaling (Krogsgaard et al., 2005). Here, soluble pMHC heterodimers—where one peptide was an agonist while the other was an endogenous self-peptide—could stimulate T cell activation and synapse formation as long as CD4 could be engaged on the agonist side of the pMHC dimer. Hence TCR dimerization may go beyond signal initiation but currently it is not clear how TCR dimerization relates to the formation of higher-order oligomers that have been described by Manz et al. (2011) and Kumar et al. (2011).

The requirement of TCR dimerization is an attractive model to explain why certain peptide binding models are not “allowed” despite distinct 3D and 2D affinities. Adams et al. compared the crystal structure of an alloreactive TCR αβ in complex with four different, but not naturally occurring, peptides all bound to the same MHC (Adams et al., 2011). While three peptides utilized germline-preserved TCR-MHC interactions and induced signaling, the fourth had a vastly different docking mode and failed to initiate signaling. The non-stimulatory peptide had a more parallel binding orientation along the α1/α2-helix of the MHC than the other stimulatory peptides. However, this cannot be the full story since an even more parallel orientation was reported for the CD1d-lipid antigen (αGalCer) of a NK TCR (Borg et al., 2007). Likewise, the 2D biophysical parameters of the non-stimulatory peptides were within the range of other agonistic pMHCs. Hence other explanations, like the formation of dimers and higher-order oligomers are needed to explain why a specific docking topology does not initiate signaling.

Ellis Reinherz proposed that selective TCR signaling may require dynamic interactions between the TCR αβ and the CD3 dimers, rather than a static on/off-switch, resulting in dynamic quaternary changes upon TCR ligation and triggering (Kim et al., 2012). In this model, the dynamic interaction between ectodomains rearranges the positioning of the CD3 dimers thereby affecting access to the cytoplasmic ITAM domains. A related dynamic model was put forward as the “safety” model of TCR triggering (Kuhns and Davis, 2008), in which electrostatic interactions sequester basic residue-rich stretches of the ITAM domains into negatively charged lipids in the inner leaflet of the plasma membrane (Aivazian and Stern, 2000; Xu et al., 2008). It was postulated that this lipid association of the cytosolic tails would prevent ITAM phosphorylation by restricting Lck access. However, ITAM phosphorylation triggered the release of these domains from the membrane (Zhang et al., 2011). This recent study also showed that mutations in the basic residue-rich stretch of CD3ζ impair TCR signaling and affect TCR localization in respect to Lck (Zhang et al., 2011). Other possible mechanisms that may dislodge ITAM motifs from the membrane are mechanical forces and changes in local membrane environment, or even a combination of the two. A change in lipid environment (which has been observed microscopically (Gaus et al., 2005) and biochemically (Zech et al., 2009) after the assembly of TCR signaling complexes) would require an initiation signal that is independent of ITAM phosphorylation. This brings us back to the question of what drives membrane restructuring and the recruitment of vesicles, such as the LAT-containing vesicles described above, and whether these processes bypasses TCR triggering. A possibility is that cell adhesion and/or the restructuring of the actin cytoskeleton trigger vesicle recruitment and fusion with the plasma membrane but how this fits into the timeline of TCR signaling and the onset of calcium fluxes within seconds of TCR triggering (Huse et al., 2007) is not clear.

## TCR as mechanosensor

Several groups have recently provided evidence that physical forces applied to the TCR or TCR subunits activate T cells, meaning that the TCR is a mechanosensor (Kim et al., 2009; Li et al., 2010; Husson et al., 2011; Judokusumo et al., 2012; Ma et al., 2012). These observations were made when beads coated with pMHC or monoclonal antibodies against CD3ε were manipulated with optical tweezers. *In vivo*, such forces could be exerted when migrating T cells attach to pMHC on APCs prior to a stop signal, or during sequential and repetitive contacts between T cells and APCs (Gunzer et al., 2000). This would mean that the affinity of pMHC-TCR interaction is translated into mechanical force, which in turn could affect the quaternary structure of the TCR/CD3 complex. Ellis Reinherz proposed that a pulling force from the pMHC causes the Cβ FG loop to push on the ectodomain of CD3ε. He speculates that multimeric crosslinking (and possibly soluble antibodies) applies a torque on the TCR to achieve the same outcome as monomeric interactions under applied mechanical force (Kim et al., 2012). If this is correct, the need for TCR dimerization could be the application of torque and the subsequent quaternary restructuring, rather than dimerization *per se*. Hence, rupture force (Husson et al., 2011) and bond lifetime under load can potentially determine the potency of pMHC stimulation. Furthermore, force on an individual TCR αβ heterodimer will be greater if fewer cognitive TCR-pMHC per cell are formed during T cell-APC contact. Hence the mechano-sensing properties of the TCR could integrate sensitivity and specificity. In adhesion biology, so-called catch bonds have been described (Marshall et al., 2003) that reinforce binding under tensile forces that expose cryptic binding sites. The characteristic of catch bond engagement is that the lifetime of bonding is no longer linear. In this context, certain pMHC-TCR interactions would be stabilized while others are not. To generate sufficient torque to expose potential catch bonds, it is likely that the TCR-pMHC docking topography is critical (Kim et al., 2009). Whether catch bonds exist in the TCR-pMHC interaction and modulate on- and off-rates and how TCR docking orientations fit in to this scenario is yet to be explored.

## Conclusion

**Figure 5:**
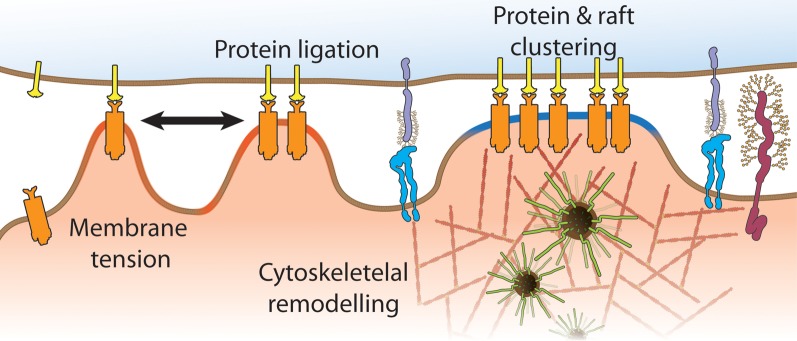
**Mechanisms that contribute to the spatial organization of TCR signaling processes include mechanical forces (black arrow), membrane tension due to convoluted membrane topography (red regions), variable inter-membrane distances across the immunological synapse, membrane compartmentalization by cortical actin and positioning and abundance of lipid domains (blue regions)**.

There is much to be learned about this pivotal immune receptor and how antigen binding initiates the assembly of multi-molecular complexes for signal initiation. What is now needed is the integration of information from TCR docking topography obtained by crystallography (Borg et al., 2007; Adams et al., 2011), to measurements and manipulations (Li et al., 2010) of mechanical forces with optical tweezers (Kim et al., 2012), biomembrane force probes (Huang et al., 2010; Husson et al., 2011) and single molecule imaging approaches (Lillemeier et al., 2010; Sherman et al., 2011; Williamson et al., 2011) that take us beyond the plasma membrane. Importantly, recent papers have given us the motivation for such cross-disciplinary work as they highlight that this uniquely complex receptor system holds the key for T cell activation (Kumar et al., 2011; Manz et al., 2011). These discoveries have given us a glimpse that the distinction between naïve and memory T cells could also lay in the spatial organization of the TCR itself. Distributions of TCR dimers, clusters and islands relative to other signaling proteins may explain why we see enhanced basal phosphorylation of LAT and ZAP70 (Kersh et al., 2003) and diminished Lck dependency (Tewari et al., 2006) in memory T cells as well as the differential activation of MAP kinases in experienced and naïve T cells (Adachi and Davis, 2011). The TCR spatial organization itself could be influence by membrane domains (Kersh et al., 2003; Tani-ichi et al., 2005), expression of adaptor proteins (Singleton et al., 2011), membrane topography (James and Vale, 2012) and applied forces (Figure 5). This will make for exciting times ahead.

### Conflict of interest statement

The authors declare that the research was conducted in the absence of any commercial or financial relationships that could be construed as a potential conflict of interest.
